# Granular honeycomb scaffolds composed of carbonate apatite for simultaneous intra- and inter-granular osteogenesis and angiogenesis

**DOI:** 10.1016/j.mtbio.2022.100247

**Published:** 2022-03-26

**Authors:** Koichiro Hayashi, Toshiki Yanagisawa, Masaya Shimabukuro, Ryo Kishida, Kunio Ishikawa

**Affiliations:** Department of Biomaterials, Faculty of Dental Science, Kyushu University, 3-1-1 Maidashi, Higashi-ku, Fukuoka, 812-8582, Japan

**Keywords:** Honeycomb, Scaffold, Bone, Granule, Extrusion molding

## Abstract

Granular porous calcium phosphate scaffolds are used for bone regeneration in dentistry. However, in conventional granules, the macropore interconnectivity is poor and has varying size. Herein, we developed a productive method for fabricating carbonate apatite honeycomb granules with uniformly sized macropores based on extrusion molding. Each honeycomb granule possesses three hexagonal macropores of ∼290 ​μm along its diagonal. Owing to these macropores, honeycomb granules simultaneously formed new and mature bone and blood vessels in both the interior and exterior of the granules at 4 weeks after implantation. The honeycomb granules are useful for achieving rapid osteogenesis and angiogenesis.

## Graphical abstract

**Figure:**
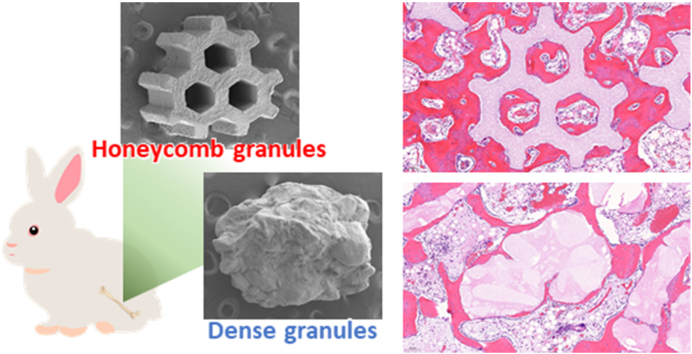

## 1 Introduction

Aging of the population is a global problem. As the population ages, bone disorders and the need for dental implants increase, which boosts the demand for bone regeneration [1,2]. Although autogenous bone grafting is the gold standard in bone regeneration, it has limitations such as limited bone quantity collection, damage to donor site, and prolonged surgery [[3], [4], [5], [6], [7]]. To resolve these problems, as an alternative to autologous bone, the development of synthetic scaffolds for bone regeneration is necessary.

Thus far, hydroxyapatite [HAp; Ca_10_(PO_4_)_6_(OH)_2_] and β-tricalcium phosphate [β-TCP; Ca_3_(PO_4_)_2_] are used as synthetic scaffolds for bone regeneration [[8], [9], [10], [11], [12]]. However, the resorption of HAp is considerably slow and it remains in the body for more than 10 years [13]. Conversely, β-TCP spontaneously dissolves and disappears before the formation of sufficient volume of new bone, causing reduced functioning of the scaffold [14]. In contrast, carbonate apatite [CAp; Ca_10-a_(PO_4_)_6-b_(CO_3_)_c_], which is a bone mineral analog in which carbonate ions substitute phosphate and hydroxyl ions in HAp, does not dissolve spontaneously and is resorbed only via osteoclastic resorption. Consequently, CAp remains and serves as the scaffold before new bone formation and then it is gradually resorbed and eventually replaced with new bone [15,16].

Despite the same chemical composition, the resorption rate and bone formation ability of the scaffold critically vary based on structure. Macroporous (>100 ​μm) and microporous (<10 ​μm) structures are dominant factors in determining responses of cells and tissues [[17], [18], [19], [20]]. Macropores of 200–300 ​μm promote both osteogenesis and angiogenesis than those <200 ​μm [21,22]. Macropores of 200–300 ​μm can prevent the penetration of fibrous tissues into the scaffold, whereas macropores >460 ​μm cannot [23]. Owing to these effects, scaffolds with macropores of 200–300 ​μm achieve superior new bone formation compared to scaffolds with other macropore sizes. Micropores affect osteoclastic resorption, followed by osteogenesis [24]. When micropore volume is 0.1–0.2 ​g/cm^3^, the scaffold resorption rate is synchronized with new bone formation rate, and consequently, the scaffold is successfully replaced with the new bone [[24], [25], [26]]. Therefore, synthetic scaffolds with precisely controlled macropore size (200–300 ​μm) and micropore volume (0.1–0.2 ​g/cm^3^) are required to achieve favorable bone regeneration.

Granular, rather than blockish, scaffolds (<2 ​mm) are used in clinical dentistry because their defect sizes in bone are usually small [[27], [28], [29], [30], [31], [32], [33], [34]]. Macroporous granules are more favorable than dense granules (DGs) for the rapid ingrowth of bone [[35], [36], [37], [38], [39], [40], [41], [42], [43], [44], [45]]. Conventionally, macroporous granules are fabricated using gas forming [35,36] and sacrificial template methods [[37], [38], [39]]. However, these methods cannot accurately control the macroporous structure. Consequently, 1) the produced granules have different sizes, number of macropores vary, and granules without macropores frequently form, 2) macropores are not interconnected and pores are closed, and 3) the macropore size within a granule varies resulting in their sizes being outside the desired range for bone regeneration (200–300 ​μm). Therefore, conventional methods allow introduction of macropores into the granule, without contributing to the promotion of bone regeneration. To resolve these problems, methods that provide accurate control of the macroporous structure should be selected for fabricating macroporous granules.

Three-dimensional (3D) printing is a useful method for fabricating scaffolds with controlled macroporous structure [[46], [47], [48]]. However, since the nozzle size of a 3D printer is a minimum of ∼300 ​μm, it is unsuitable for the fabrication of macroporous granules.

Here, we describe the highly productive fabrication of CAp honeycomb granules (HCGs) with precisely shape-controlled macropores of 200–300 ​μm via extrusion molding. We describe the osteogenesis and angiogenesis abilities of HCGs with micropores by comparison with CAp dense granules (DGs) with the same micropore volume as that of the HCGs.

## 2 Materials and methods

### 2.1 Fabrication of HC green body granules

HC green body granules were fabricated using the extrusion molding method modified from our previous report [21]. A mixture of CaCO_3_ powder (Sakai Chemical Industry, Osaka, Japan) and an acryl resin-based binder (Nagamine Manufacturing, Kagawa, Japan) were extruded with an extruder equipped with a die with a slit thickness of 300 ​μm and a pitch of 600 ​μm (Lab Plastmill, Toyo Seiki Seisaku-sho Ltd., Tokyo, Japan). As a result, HC green bodies were extruded on a conveyor belt. The HC green bodies were cut with a guillotine cutter into 0.6–1 ​mm-long granules.

### 2.2 Fabrication of CAp HCGs

HC green body granules were subjected to heat treatment at 600 ​°C for 24 ​h for removing the organic binder and mildly sintering the granules. After the heat treatment, CaCO_3_ HCGs were obtained. The calcium carbonate HCGs were immersed in a 1 ​mol/L Na_2_HPO_4_ solution (FUJIFILM Wako Pure Chemical Corporation, Osaka, Japan) at 80 ​°C for 7 ​d. As a result, CaCO_3_ HCGs were phosphatized through dissolution–precipitation reactions, and the chemical composition was changed to that of CAp. Finally, the obtained CAp HCGs were washed 10 times with distilled water.

### 2.3 Physicochemical and mechanical characteristics of CAp HCGs

X-ray diffraction patterns of HCGs and DGs (GC Corporation, Tokyo, Japan) were measured to determine the crystal phases using an X-ray diffractometer (XRD; D8 Advance, Bruker AXS GmbH, Karlsruhe, Germany). The functional groups in the HCGs and DGs were determined using a Fourier transform infrared (FTIR) spectrophotometer (FT-IR-6200, JASCO, Tokyo, Japan). HAp (Taihei Chemical Industrial, Osaka, Japan) was used as the reference. The percentages of carbonate contained in the HCGs were measured using an elemental CHN analyzer (MT-6, Yanako Analytical Instruments, Kyoto, Japan). The average percentages of carbonate contained in the HCGs were calculated from the values of eight samples per group. The microstructures of the HCGs and DGs were observed using a scanning electron microscope (SEM; S3400 ​N, Hitachi High-Technologies, Tokyo, Japan). The porous characteristics of the HCGs and DGs were measured using mercury intrusion porosimetry (MIP, AutoPore 9420, Shimadzu Corporation, Kyoto, Japan). The loads applied to HCGs and DGs at their failures were measured through the uniaxial compression test using a universal testing machine (Autograph AGS-J, Shimadzu Corporation, Kyoto, Japan). The average compression loads were calculated from the results of six granules per group.

### 2.4 Weight loss and release of calcium and phosphate ions in buffer solutions

HCGs and DGs (50 ​mg) were immersed in 20 ​mL of 0.05 ​mol/L tris(hydroxymethyl)aminome thane–HCl buffer solution (pH 7.4, corresponding to physiological pH) and 0.08 ​mol/L acetic acid–sodium acetate solution (pH 5.5, corresponding to a weak acidic environment due to the acids produced by osteoclasts). After 3 ​h and 1, 3, and 7 ​d of immersion, the granules and supernatants were collected. The granules were washed with distilled water 10 times and dried at 100 ​°C overnight. The weights of the dried granules were measured, and the weight losses were calculated. The concentrations of calcium and phosphate ions released from HCGs and DGs in the supernatants were measured using inductively coupled plasma optical emission spectrometry (ICP-OES) based on Optima 7300 DV (PerkinElmer, MA, USA).

### 2.5 In vitro cell proliferation

The cytotoxicity of HCGs and DGs was evaluated by assessing cell proliferation using a water-soluble tetrazolium (WST) salt (n ​= ​6). Human umbilical cord mesenchymal stem cells were purchased from the JCRB Cell Bank (Osaka, Japan). The cells were seeded in a 24-well plate, at 1.0 ​× ​10^5^ ​cells/well, and cultured in a culture medium (Plusoid-M, GlycoTechnica, Kanagawa, Japan) for 24 ​h at 37 ​°C in a humidified atmosphere containing 5% CO_2_. Then, the HCG and DG-containing Falcon® cell culture inserts from the 24-well plate with 0.4 ​μm transparent polyethylene terephthalate membrane (Corning, NY, USA) were set at each well. After 6 ​h, 24 ​h, 48 ​h, and 7 ​d of culture, cell number per well was assayed using the WST-8 assay kit (DOJINDO, Kumamoto, Japan). The cytotoxicity of HCGs and DGs was evaluated via a comparison with the cell number per well cultured without materials, i.e., the control group.

### 2.6 Ethics statement

All experiments involving animals were conducted according to the ethical policies and procedures approved by the Animal Care and Use Committee of Kyushu University, Japan (Approval No. A30-237-0; issued August 1, 2018).

### 2.7 Animals

Japanese white rabbits (18 weeks of age, 3.0–3.5 ​kg of body weight) were purchased from Japan SLC (Shizuoka, Japan) for *in vivo* evaluations. These rabbits were single-housed in cages and maintained on a standard diet with an adequate amount of water at the Center of Biomedical Research, Research Center for Human Disease Modeling, Graduate School of Medical Sciences, Kyushu University. In total, 6 rabbits were used (n ​= ​6 per group).

### 2.8 Surgical procedure

The rabbits were subjected to an intramuscular injection of anesthesia composed of combined xylazine (5.0 ​mg/kg) and ketamine (30 ​mg/kg). The femur fur of the rabbit was shaved in both legs. The femoral skin was disinfected with 10% w/v povidone–iodine (Meiji Seika Pharma, Tokyo, Japan). The femur condyle was exposed by making an incision in the femoral skin (approximately 2 ​cm in length) using a scalpel. The periosteum was separated from the bone using a raspatorium. HCGs and DGs were separately implanted into the critical size defects (6 ​mm in diameter and 3 ​mm in depth) produced in the femur condyles of both legs. The periosteum and subsequently the incised skin were sutured. Finally, the surgical site was disinfected with 10% w/v povidone–iodine and gentamicin sulfate solution (Gentacin®, Takata Pharmaceutical, Saitama, Japan) was intraperitoneally injected for preventing infection.

### 2.9 In vivo analyses

At week 4 after the implantation of the HCGs and DGs, the rabbit femurs (n ​= ​6 per group) were collected and immersed in formalin solution for fixing them. The μ-computerized tomography (μ-CT) images of the bone defect region were obtained by μ-CT scanning (SkyScan, Bruker Corporation, Billerica, MA, USA) using the collected specimens. After μ-CT scanning of the specimens, these were decalcified, embedded in paraffin, and cut to obtain the sections. The sections were treated with hematoxylin and eosin (HE), tartrate-resistant acid phosphatase (TRAP), and Masson's trichrome (MT) staining. Regions stained blue by MT corresponded to collagen fibers in the bone. The histological images of the HE-, TRAP-, and MT-stained tissue sections were obtained using a microscope (BZ-X, Keyence, Osaka, Japan). The percentages of new bone and remaining materials and the number of osteoclasts were estimated using HE-, MT-, and TRAP-stained sections with the BZ-X digital analysis software.

### 2.10 Statistical analysis

All data are presented as mean ​± ​standard deviation. *p*-values < 0.05 were considered statistically significant. Comparisons between groups were performed using Student's t-test.

## 3 Results

**Fig. 1:**
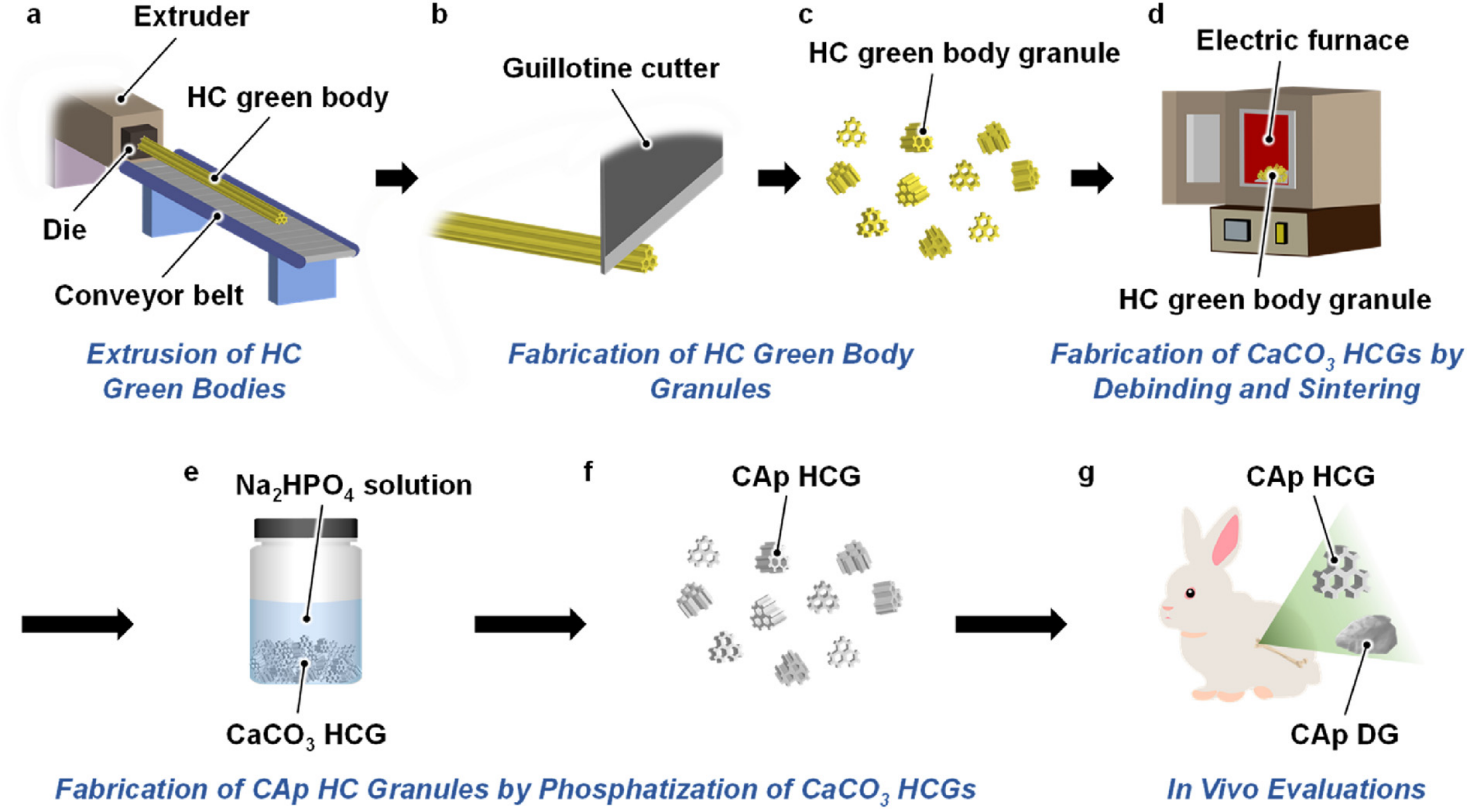
Schematic illustration for fabrication of CAp HCGs and animal experiments. (a) Extrusion of HC green bodies, (b) cutting of the HC green bodies (c) to obtain HC green body granules. (d) Heat treatment of the HC green body granules to obtain CaCO_3_ HCGs via debinding and sintering. (e) Phosphatization of CaCO_3_ HCGs by immersing them in an Na_2_HPO_4_ solution (f) to obtain CAp HCGs. (g) Implantation of CAp HCGs and DGs into a critical size bone defect produced in the rabbit femur condyle.

HC green bodies were continuously extruded on a conveyor belt (Fig. 1a) and subsequently cut at 0.6–1-mm intervals using a guillotine cutter (Fig. 1b), thereby obtaining granules (Fig. 1c). In a previous study, we removed the organic binder from the HC green bodies by heating above 600 ​°C for 24 ​h [21,49,50]. HC green bodies were modestly sintered and their microporosity was kept at 0.1–0.2 ​cm^3^/g by heating at 600 ​°C for 24 ​h [21]. In this study, accordingly, HC green body granules underwent debinding, i.e., removal of the organic binder, and modest sintering at 600 ​°C for 24 ​h (Fig. 1d). The obtained CaCO_3_ HCGs were immersed in an Na_2_HPO_4_ solution at 80 ​°C for 7 ​d to convert the chemical composition into that of CAp (Fig. 1e) through a dissolution-precipitation reaction as follows [26]:

(1) CaCO_3_ → Ca^2+^ ​+ ​CO_3_^2−^

(2) Ca^2+^ ​+ ​PO_4_^3−^ ​+ ​CO_3_^2−^ → Ca_10-a_(PO_4_)_6-b_(CO_3_)_c_

Thus, CaCO_3_ was dissolved in a phosphate salt aqueous solution, and calcium and carbonate ions were released in the solution [eq. (1)]. In the resultant solution, CAp precipitated owing to its supersaturation [eq. (2)]. These reactions gradually occurred from HCG surface to the center. Consequently, the entire shape of HCGs was maintained, although its chemical composition changed. CAp HCGs were obtained after washing with distilled water several times (Fig. 1f). For *in vitro* and *in vivo* evaluation, CAp HCGs were subjected to dry-heat sterilization at 170 ​°C for 3 ​h. The sterilized CAp HCGs were implanted into a critical-size bone defect in the medial condyle of rabbit femur (Fig. 1g). CAp DGs, used as the control scaffolds, were also sterilized and implanted using the same methods as for CAp HCGs.

**Fig. 2:**
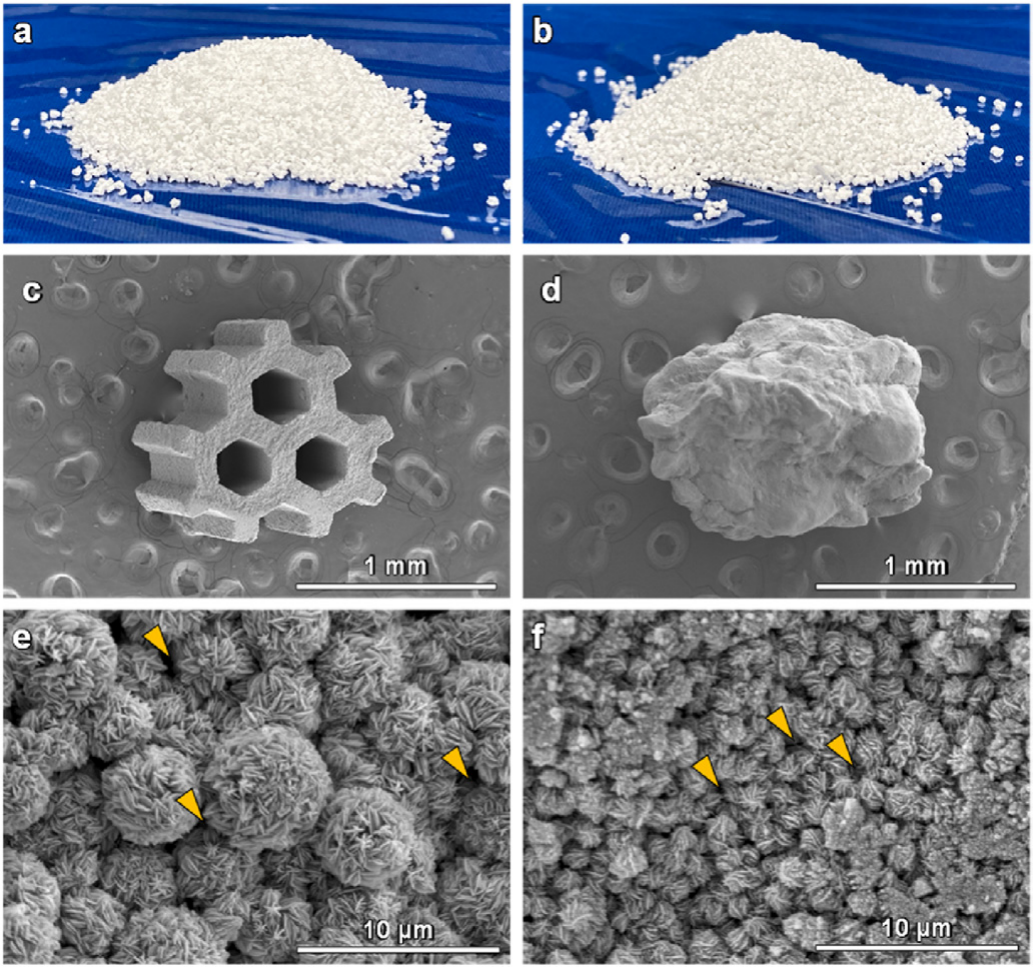
Photographs of (a) CAp HCGs and (b) DGs. SEM images of (c) CAp HCGs and (d) DGs. High-magnification SEM images of (e) CAp HCGs and (f) DGs.

Both CAp HCGs (Fig. 2a) and DGs (Fig. 2b) were produced in adequate quantity. CAp HCGs possessed three hexagonal macropores (Fig. 2c). The granular size was 1270 ​± ​68 ​μm and the diagonal line length of the hexagonal macropores was 288.7 ​± ​3.5 ​μm (Fig. 2c). CAp DGs had no macropores and the granular sizes in the long and short axis directions were 1399 ​± ​45 and 1096 ​± ​17 ​μm, respectively (Fig. 2d). Thus, the granular size of CAp HCGs was almost equal to that of DGs. The higher magnification images showed that both CAp HCGs and DGs had micropores among spherical CAp crystal aggregates (Fig. 2e and f). Furthermore, the cross-section images clarified that the macropores penetrated the CAp HCGs (Fig. S1a) and the microstructure of HCG interior was similar to that of the surface (Fig. S1b and c). In contrast, the DGs had no macropores (Fig. S1d), and DG microstructure differed between the surface and interior (Fig. S1e and f). Since the dissolution-precipitation reactions [Eqs. (1), (2)] inevitably occur from the surface to the interior, the difference in granular microstructure between the surface and interior was considered to be caused by the variations in the permeability of the phosphate salt aqueous solution into the granules. The macropores in the HCGs facilitated the permeability of phosphate salt aqueous solution into the granules, endowing the HCGs with a surface-like interior microstructure.

**Fig. 3:**
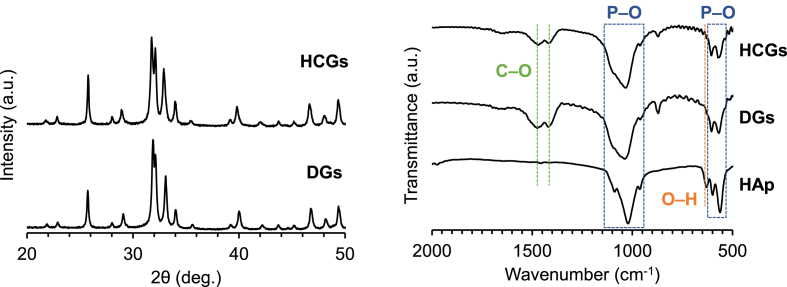
(a) XRD patterns of CAp HCGs and DGs. (b) FTIR spectra of CAp HCGs, DGs, and HAp powder.

The XRD patterns of HCGs and DGs corresponded to that of an apatitic calcium phosphate such as HAp (Fig. 3a). In FTIR spectra of HCGs, DGs, and HAp, the phosphate absorption bands appeared at 611–534 and 1141–917 ​cm^−1^ (Fig. 3b) [51,52]. Although the absorption band attributed to hydroxyl was observed at 626 ​cm^−1^ in the HAp spectrum [52], the hydroxyl bands were absent in the spectra of HCGs and DGs [51]. In contrast, the doublet bands corresponding to carbonate appeared at 1551–1330 ​cm^−1^ in the spectra of HCGs and DGs, whereas the doublet bands were not present in the spectrum of HAp [51]. The doublet bands at 1471 and 1415 ​cm^−1^ were attributed to carbonates that substituted for hydroxyl and phosphate in HAp. These XRD and FTIR results demonstrated that HCGs and DGs were composed of AB-type CAp [51]. The CHN analysis demonstrated that the carbonate contents in the HCGs were 7–9%, which coincided with the carbonate content in human bone apatite [53].

**Fig. 4:**
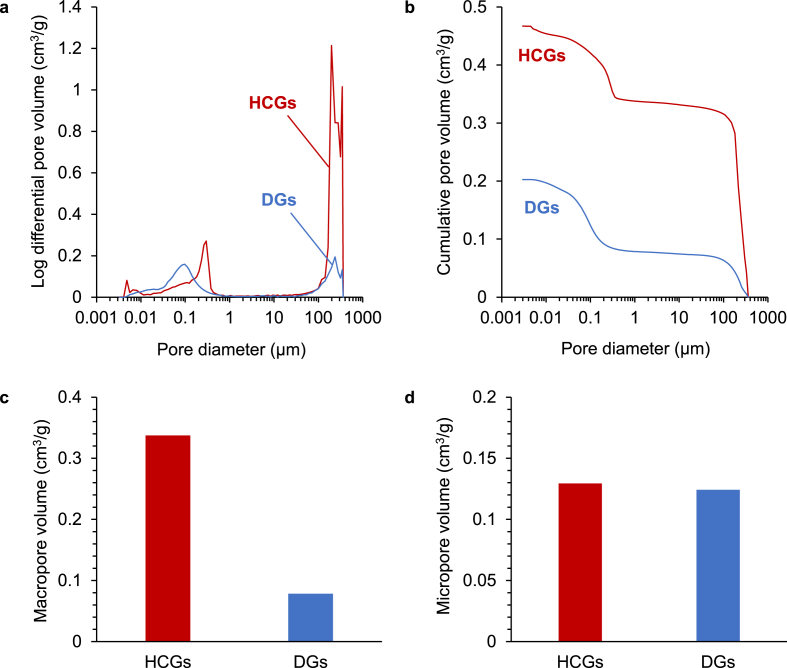
Porous structure characteristics of CAp HCGs and DGs measured via mercury intrusion porosimetry. (a) Pore size distribution and (b) cumulative pore volume versus pore size. Volumes of (c) macropores and (d) micropores.

The MPI curves of both HCGs and DGs (Fig. 4a) showed peaks in both regions of macropores (>100 ​μm) and micropores (<10 ​μm). In the MPI curve of HCGs, the peaks in the macropore region were attributed to combined intragranular macropores and intergranular spaces. In contrast, in the MPI curve of DGs, the peaks in the macropore region were ascribed merely to intergranular spaces. Therefore, the HCGs possessed ∼4-fold larger pore volume in the macropore region than DGs: the macropore region pore volumes in the HCGs and DGs were 0.34 and 0.08 ​cm^3^/g, respectively (Fig. 4b and c). In contrast, both the HCGs and the DGs had 0.12 ​cm^3^/g of micropore volume, thus their micropore volumes were equal (Fig. 4b and d).

**Fig. 5:**
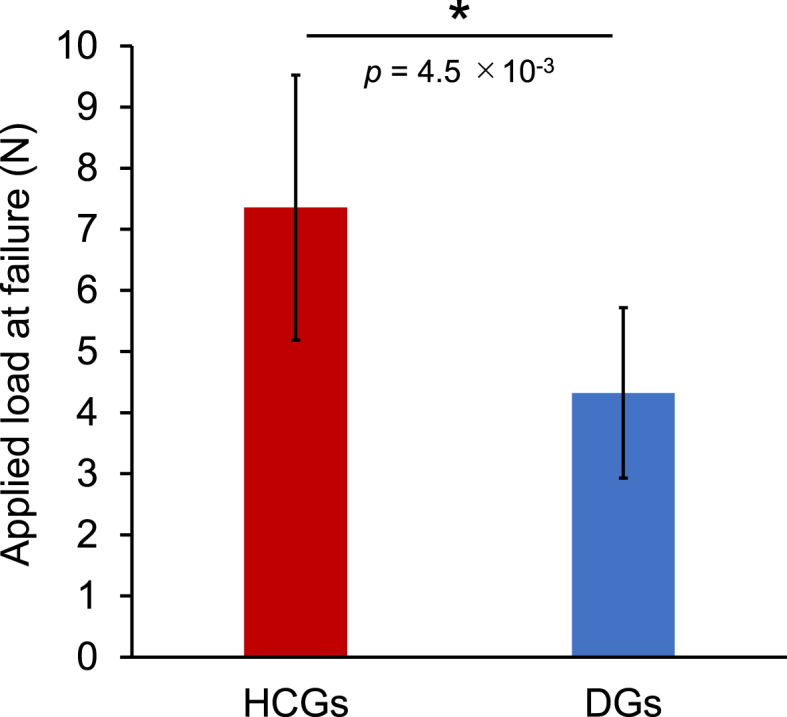
Compression load applied to HCGs and DGs at failure. ∗*p* ​< ​0.01.

The applied load at failure of HCGs and DGs was 7.4 ​± ​2.2 and 4.3 ​± ​1.4 ​N, respectively (Fig. 5). Thus, the HCGs showed significantly higher resistance to compression than the DGs (*p* ​= ​4.5 ​× ​10^−3^). This is probably because the shape of DGs was irregular and they bore compression load at a specific point, whereas the HCGs had flatter surfaces than DGs and they could bear load on the face. Thus, the granular shape rather than the honeycomb structure may highly affect the mechanical strength.

**Fig. 6:**
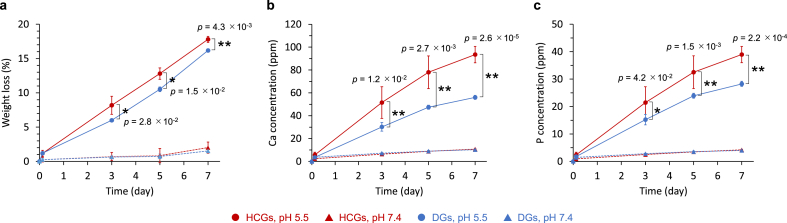
(a) Weight losses of HCGs and DGs in buffer solutions at pH 7.4 and 5.5. (b) Calcium and (c) phosphate ions released from HCGs and DGs in buffer solutions at pH 7.4 and 5.5. ∗*p* ​< ​0.05 and ∗∗*p* ​< ​0.01.

A small percentage (∼2 ​wt%) of HCGs and DGs was dissolved in physiological saline (pH 7.4) for 7 ​d, whereas they were dissolved in large quantity (above 15 ​wt%) under a weak acidic environment (pH 5.5) resulting from the acids produced by osteoclasts (Fig. 6a). Consequently, larger amounts of calcium (Fig. 6b) and phosphate ions (Fig. 6c) were released at pH 5.5 than at pH 7.4 during the immersion period of 7 ​d. Furthermore, the amounts of calcium and phosphate ions released from the HCGs were significantly higher than those from the DGs (Fig. 6b and c). The differences in ion release between the HCGs and DGs were considered to be caused by the differences in surface area.

**Fig. 7:**
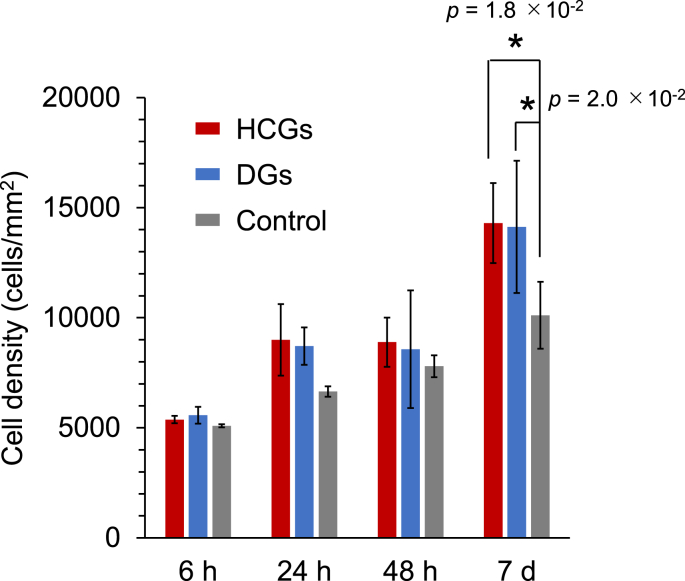
Cell proliferation assays during culture period of 7 ​d ∗*p* ​< ​0.05.

The cytotoxicity of HCGs and DGs was evaluated by assaying cell proliferation. The cell number per well in the HCG and DG groups increased with culture period (Fig. 7). After 7 ​d of cell culture, the cell number per well in the HCG and DG groups was significantly higher than that in the control group (*p* ​= ​1.8 ​× ​10^−2^ and 2.0 ​× ​10^−2^, respectively). Small amounts of calcium and phosphate ions were released from HCGs and DGs at natural pH (Fig. 6b and c), probably promoting cell proliferation [54].

**Fig. 8:**
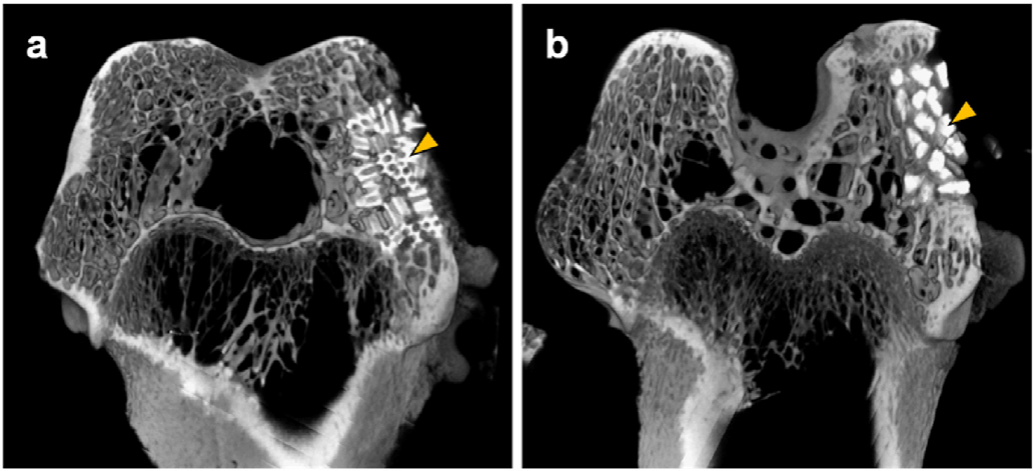
μ-CT images at week 4 after implantation of CAp HCGs (a) and DGs (b) into the rabbit femur defect. Yellow arrowheads indicate the HCGs and DGs.

HCGs and DGs were implanted into the bone defect in the rabbit femoral condyle. The μ-CT in the HCGs-implanted group showed that the HCGs remained in the defect and new bone formed in intragranular macropores and intergranular spaces at week 4 post-implantation (Fig. 8a). The DGs also remained in the defect (Fig. 8b). Although new bone formed in intergranular spaces, it did not form within the granules (Fig. 8b). Since the partially resorbed regions in these granules and new bone region were not clearly distinguished, quantification of new bone percentage was difficult by μ-CT.

**Fig. 9:**
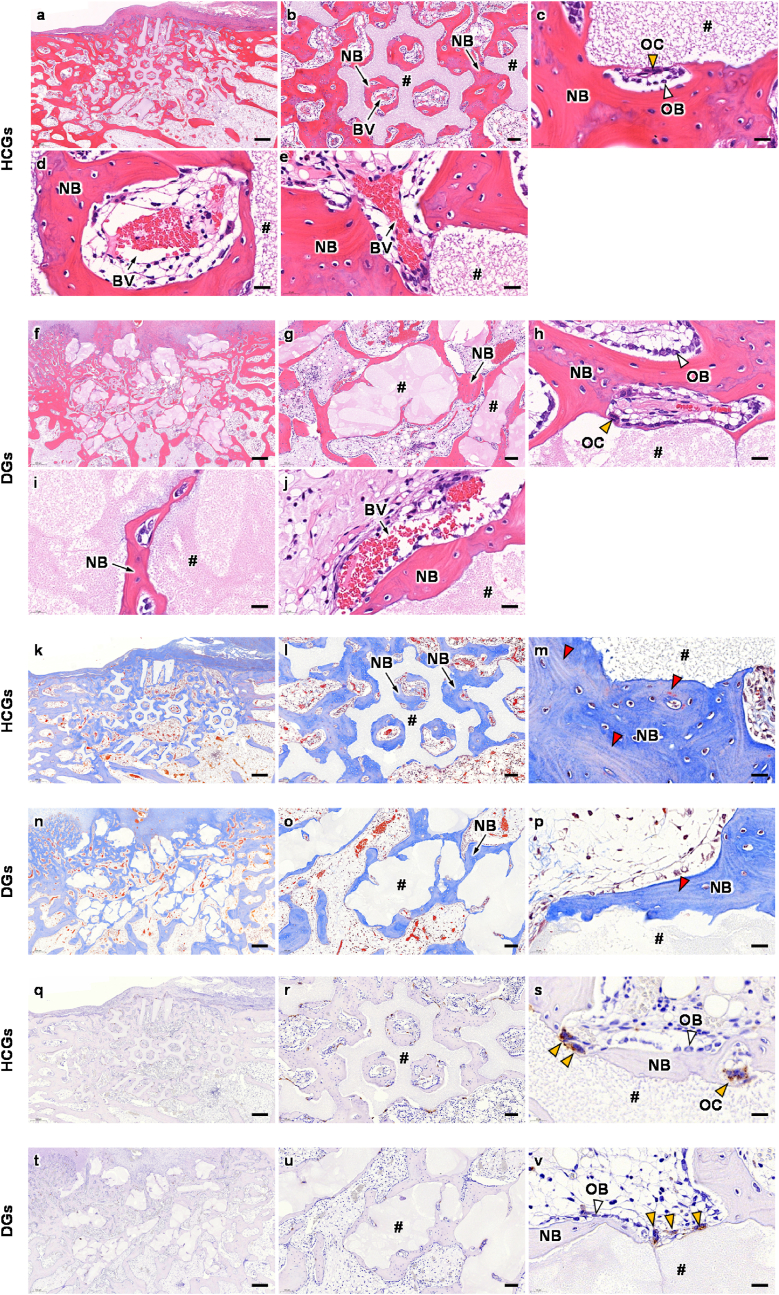
(a–j) HE-, (k–p) MT-, and (q–v) TRAP-stained sections at 4 weeks after implantation of HCGs and DGs. Panels b, g, l, o, r, and u are magnification images of panels a, f, k, n, q, and t, respectively. Panels c, h, m, p, s, and v are higher magnification images for showing granular surfaces. Panels d and i are higher magnification images for showing tissues formed in intragranular (d) macropores and (i) cracks. Panels e and j are higher magnification images for showing tissues formed in the intergranular spaces. NB, BV, OB, OC, and # indicate new bone, blood vessel, osteoblast, osteoclast, and remaining granule, respectively. White, yellow, and red arrowheads indicate osteoblast, osteoclast, and new mature bone, respectively. Scale bars: (a, f, k, n, q, and t) 500 ​μm; (b, g, l, o, r, and u) 100 ​μm; and (c–e, h–j, m, p, s, and v) 20 ​μm.

To quantify the new bone percentage and reveal the responses of cells and tissues, histological analyses were conducted (Fig. 9). HE-stained sections in the HCGs-implanted group showed that new bone formed in both intragranular macropores and intergranular spaces (Fig. 9a and b) and osteoblasts and osteoclasts resided on new bone and the granule surface, respectively (Fig. 9c), at week 4 PI. Blood vessels were formed in both intragranular macropores of the HCGs (Fig. 9b and d) and intergranular spaces (Fig. 9b and e). In the DGs-implanted group, since the DGs possessed no intragranular macropores, the new bone mainly formed in intergranular spaces, although a small quantity of the new bone also formed in cracks generated in the DGs (Fig. 9f and g). Osteoblasts and osteoclasts were present on new bone and the DG surface, respectively (Fig. 9h), which was similar to the findings in the HCGs-implanted group. Blood vessels hardly formed in cracks generated within the DGs (Fig. 9i), and they formed only in the intergranular spaces (Fig. 9j). MT-stained sections showed that collagen fibers in bone were stained blue in both HCGs- and DGs-implanted groups (Fig. 9k‒p). New bone formed in both intragranular macropores and intergranular spaces in the HCGs-implanted group (Fig. 9k and l), whereas new bone formed in intergranular spaces merely in the DGs-implanted group (Fig. 9n and o). These findings were coincident with the findings in HE-stained sections. In both these groups, new bones showed a lamellar structure (Fig. 9m and p) and demonstrated that they were mature. TRAP-stained sections showed that the osteoblast resided on the HCGs (Fig. 9q–s) and DGs (Fig. 9t–v), which coincided with the findings in HE-stained sections.

**Fig. 10:**
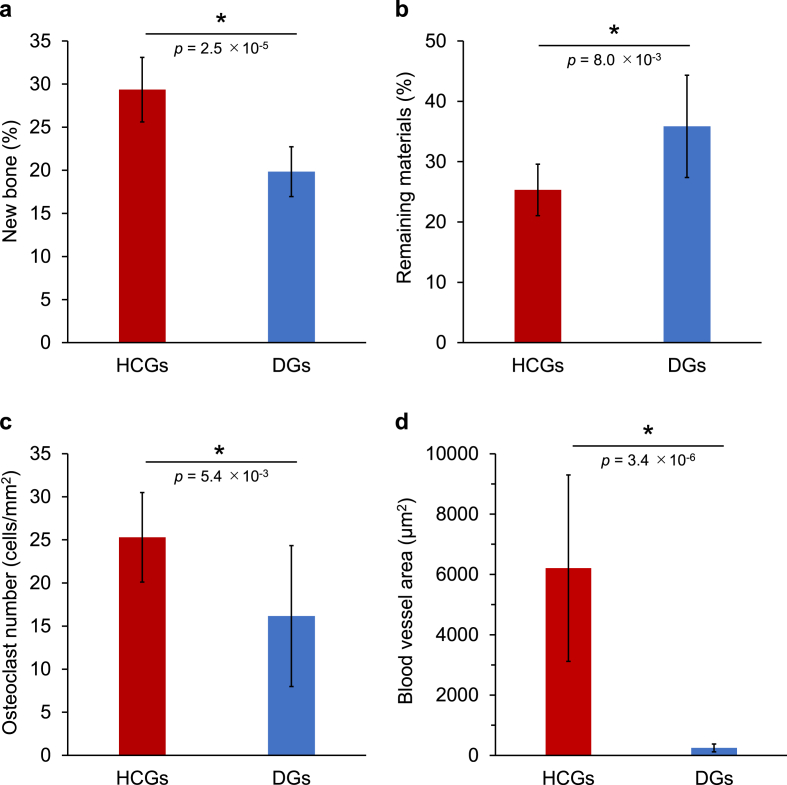
Percentages of (a) new bone and (b) remaining materials, (c) the number of osteoclasts on granules, and (d) areas of blood vessels formed in the granules. ∗*p* ​< ​0.01.

The percentages of new bone (Fig. 10a) and remaining materials (Fig. 10b) and the numbers of osteoclasts (Fig. 10c) in the defect were quantified by analyses using the HE, MT, and TRAP-stained sections. The new bone percentages in the HCGs- and DGs-implanted groups were 29.4 ​± ​3.7 and 19.8 ​± ​2.9%, respectively (Fig. 10a). The HCGs formed new bone in both the granular interior and surface simultaneously owing to the intragranular macropores, whereas the DGs formed new bone merely on the granular surface. Consequently, the HCGs formed significantly larger volume of new bone than the DGs (*p* ​= ​2.5 ​× ​10^−5^). The percentages of remaining HCGs and DGs in the defect were 25.3 ​± ​4.3% and 35.9 ​± ​8.5%, respectively (Fig. 10b). Thus, the percentage of remaining HCGs was significantly lower than that of remaining DGs (*p* ​= ​8.0 ​× ​10^−3^). This result was reasonable because the HCGs possessed intragranular macropores and the original granular volume of the HCGs was lower than that of the DGs. The osteoclast numbers in the HCGs and DGs-implanted groups were 25.3 ​± ​5.2 and 16.2 ​± ​8.2 ​cells/mm^2^, respectively (Fig. 10c), showing significantly different (*p* ​= ​5.4 ​× ​10^−3^). This was presumed to be caused by the difference in surface area between the HCGs and DGs. Owing to the intragranular macropores, the HCGs had a larger surface area than that of the DGs. The areas of blood vessels formed in HCGs and DGs were 6208 ​± ​3089 and 251 ​± ​131 ​μm^2^, respectively (Fig. 10d). Thus, the area of blood vessels formed in HCGs was significantly larger than that in DGs (*p* ​= ​3.4 ​× ​10^−6^).

## 4 Discussion

This study established a fabrication method based on extrusion molding for mass production of HCGs with precisely controlled size, shape, and macroporous structure. When macroporous granules are fabricated by conventional methods such as gas forming [35,36] and sacrificial template methods [[37], [38], [39]], most of the macropores are present as closed pores and their interconnectivity is poor. Even in interconnected macropores, the connecting parts between macropores become smaller than that of the macropores in principle and their sizes are difficult to control. Therefore, the size of the macropore varies from region to region within the granule and in spots it falls outside the effective size range for promoting osteogenesis (200–300 ​μm). In contrast, in the HCGs, all intragranular macropores completely penetrate the granules and their sizes are constant within the granule and are effective for osteogenesis. Thus, the proposed method resolves problems in conventional methods and produces novel type macroporous granules, i.e., HCGs, for achieving rapid bone regeneration.

The HCGs form the new bone simultaneously in the interior and exterior of the granules owing to the precisely controlled macroporous structure, whereas DGs form the new bone merely on the surface, except a small quantity of bone that is formed within cracks generated in the granules. Furthermore, the intragranular macropores increase the granular surface area, i.e., area for cellular attachment, allowing larger number of osteoclasts to reside on HCGs than on DGs. Thereby, the HCGs are more easily subjected to osteoclastic resorption and release larger amounts of calcium and phosphate ions than DGs. Thus, the bone volume in the HCGs-implanted group was 1.5-fold larger than that in the DGs-implanted group. The micropore volumes in the HCGs were equal to those in the DGs and were within the favorable range for bone regeneration. Thus, the effect of macropores alone increases new bone volume 1.5-fold.

Furthermore, all HCGs formed a number of large blood vessels within the intragranular macropores, whereas a few DGs formed small blood vessels in cracks generated in the granules. The area of blood vessels formed in HCG intragranular macropores was ∼25-fold larger than that in DG cracks. Thus, the intragranular macropores contribute substantially to both osteogenesis and angiogenesis within granules, whereas cracks generated during the process of granule resorption cannot serve as a substitute for intragranular macropores.

In the calcium phosphate granules fabricated by the conventional methods, soft tissues and immature bone were formed within the intragranular macropores at week 4 post-implantation (PI) [37,38] and no tissues were formed in some macropores even at day 90 [35]. In contrast, in the present study, new bone had already formed and matured in all intragranular macropores of the HCGs. In addition, blood vessels were also surrounded by new bone in the intragranular macropores of the HCGs, whereas they were absent in the macropores of macroporous granules fabricated by the conventional methods. Thus, the present study revealed that macropores of HC in the size range of 200–300 ​μm promote the ingrowth of both blood vessels and bone in the set and facilitate bone maturation in the HCGs.

Although the present study demonstrated the superior osteogenic and angiogenic abilities of HCGs within a short period after implantation, long-term results couldn't be evaluated. Nevertheless, we have previously demonstrated the long-term results of CAp blockish scaffolds in the same animal model as in this study [24,55]. The number of osteoclasts on CAp blockish scaffolds increased as their micropore volume increased [24,55], and the number of osteoclasts decreased remarkably during 8 weeks, from 4 weeks PI to 12 weeks PI [24,55]. For example, on honeycomb blockish scaffolds with 0.15 ​cm^3^/g of micropore volume, osteoclast number was 30.7 ​± ​5.9 ​cells/mm^2^ at 4 weeks PI and 10.9 ​± ​9.0 ​cells/mm^2^ at 12 weeks PI [24]. Furthermore, on multiscale porous blockish scaffolds made of CAp HCGs with 0.15 ​cm^3^/g of micropore volume, osteoclast number was 31.6 ​± ​9.5 ​cells/mm^2^ at 4 weeks PI and 16.3 ​± ​5.2 ​cells/mm^2^ at 12 weeks PI [54]. These blockish scaffolds achieved lasting replacement with new bones throughout 12 weeks PI and formed larger bones than other scaffolds with larger (0.18 ​cm^3^/g) and smaller (0.07 ​cm^3^/g) volumes of micropores at both 4 weeks PI and 12 weeks PI [24]. In this study, the micropore volume of HCGs was 0.12 ​cm^3^/g and the osteoclast number on the HCGs was 25.3 ​± ​5.2 ​cells/mm^2^ at 4 weeks PI, which is consistent with the above-mentioned findings on the blockish scaffolds. Thus, the replacement of HCGs with the new bone is expected to be maintained from 4 weeks PI to 12 weeks PI.

## 5 Conclusion

CAp HCGs with precisely controlled size, shape, and macroporous structure were effectively fabricated using an extrusion molding method. All hexagonal macropores of ∼290 ​μm size completely penetrated the HCGs. The HCGs formed the new bone and blood vessels simultaneously in the interior and exterior of the granules owing to the precisely controlled macroporous structure. In contrast, DGs formed the new bone merely on the surface. Consequently, the bone volume in the HCGs-implanted group was 1.5-fold larger than that in the DGs-implanted group.

## Credit author statement

Koichiro Hayashi: Conceptualization, Methodology, Investigation, Resources, Writing – original draft, Writing – review & editing, Visualization, Supervision, Project administration, Funding acquisition; Toshiki Yanagisawa: Investigation; Masaya Shimabukuro: Investigation; Ryo Kishida: Investigation; Kunio Ishikawa: Writing – review & editing, Project administration, Funding acquisition.

## Funding

This study was supported by the 10.13039/100009619AMED [Grant Numbers JP21im0502004h and JP21he0422005j] and 10.13039/501100001691JSPS [Grant Number JP19K22970 and JP21K17010].

## Data availability statement

Data are available on request from the authors.

## Declaration of competing interest

The authors declare that they have no known competing financial interests or personal relationships that could have appeared to influence the work reported in this paper.
