# Supplementary material for: Granular honeycomb scaffolds composed of carbonate apatite for simultaneous intra- and inter-granular osteogenesis and angiogenesis
Source: Mater Today Bio. 2022 Mar 26;14:100247. doi: 10.1016/j.mtbio.2022.100247 (PMC8976130; doi:10.1016/j.mtbio.2022.100247)
Supplement: Multimedia component 1 [file mmc1.docx]

**Supplemental materials**

Fig. S1. SEM images of cross-sections of (a-c) HCGs and (d-f) DGs. (b, e) Magnification images of panels a and d, respectively. (c, f) Magnification images of panels b and e, respectively. Yellow arrowheads indicate micropores. Scale bars: (a, d) 500 μm, (b, e) 100 μm, and (c, f) 20 μm.
